# Effect of X-rays on transcript expression of rat brain microvascular endothelial cells: role of calcium signaling in X-ray-induced endothelium damage

**DOI:** 10.1042/BSR20193760

**Published:** 2020-04-28

**Authors:** Qibing Wu, Yang Fang, Xinchen Zhang, Fei Song, Yang Wang, Hongbo Chen, Juan Du, Chang-Bo Zheng, Bing Shen

**Affiliations:** 1 Department of Radiotherapy, The First Affiliated Hospital of Anhui Medical University, Hefei, Anhui 230032, China; 2 School of Basic Medical Sciences, Anhui Medical University, Hefei, Anhui 230032, China; 3 Anhui Province Key Laboratory of Reproductive Health and Genetics, The First Affiliated Hospital of Anhui Medical University, Hefei, China; 4 Department of Obstetrics and Gynecology, Maternal and Child Health Hospital Affiliated to Anhui Medical University, 15 Yimin Road, Hefei, China; 5 School of Pharmaceutical Science and Yunnan Key Laboratory of Pharmacology for Natural Products, Kunming Medical University, Kunming, Yunnan 650500, China

**Keywords:** brain microvascular endothelial cell, Ca2+ signal, radioactive brain edema, RNA-Seq

## Abstract

Radiation-induced brain edema is a serious adverse effect of radiotherapy. Although there are many causes of radiation-induced brain edema, the pathogenesis is not clear and clinical treatment is not ideal. Therefore, knowing the differential expression of the brain microvascular endothelial cell (BMEC) transcriptome after brain radiotherapy may shed light on the pathogenesis of radiation-induced brain edema. The present study used RNA-Seq technique to identify 383 BMEC transcripts differentially expressed (many 2-fold or higher; *P* < 0.05) between control and X-ray–treated primary cultured rat BMECs. Compared with controls, X-ray–treated BMECs had 183 significantly up-regulated transcripts and 200 significantly down-regulated transcripts. The differentially expressed genes were associated with the biological processes of the cell cycle, apoptosis, vascular permeability, and extracellular junctions. The functional changes identified in the X-ray–treated BMECs included Ca^2+^ signaling, phosphoinositide 3-kinase–Akt signaling, and methionine degradation. These results indicated that transcript expression was substantially affected by radiation exposure and the proteins encoded by these differentially expressed genes may play a significant role in radiotherapy-induced brain edema. Our findings provide additional insight into the molecular mechanisms of radiation-induced brain edema and may be helpful in the development of clinical treatment of this adverse reaction to radiotherapy.

## Introduction

Radiation therapy is one of the most common treatments for malignant tumors of the head and neck. It greatly benefits patients that have nasopharyngeal carcinoma, primary brain tumor, or brain metastases [1], in particular, radiation therapy is currently the preferred treatment for patients with nasopharyngeal carcinoma [2]. However, recent clinical studies have found that brain microvascular endothelial cell (BMEC) damage and radiation-induced brain edema are common in patients with intracranial tumors after radiotherapy [3]. Radiation-induced cerebral edema may cause significant decrease in the utilization of glucose by brain tissue, leading to ischemia and hypoxia, which are the main causes of the exacerbation of clinical symptoms and neurological deficits [4,5]. The current treatment of radiation-induced brain injury in clinical practice is challenging. Despite many studies investigating radiation-induced brain injury, its pathogenesis is still unclear [6].

At present, it is believed that BMECs are highly sensitive to radiation and may be the most important effector cells of radiation-induced cerebral edema [7,8]. The apoptosis or death of BMECs can directly cause capillary structure abnormalities and blood–brain barrier (BBB) disintegration [9]. Intercellular junction types include tight junctions, desmosomes, adherens junctions, and gap junctions [10]. Tight junctions consist primarily of intrinsic transmembrane proteins, scaffold proteins, and regulatory molecules between cells [10]. Studies have shown that tight junctions between vascular endothelial cells play a critical role in the BBB [11]. Thus, most studies on the effects of radiation on blood vessels have focused on endothelial cells [7].

High-throughput technology enables comprehensive monitoring of biological systems and provides an entry point to clarify molecular mechanisms that are involved in cell responses to environment changes. Therefore, we investigated the effect of a dose of X-rays (20 Gy) on transcripts in rat BMECs [11,12] to provide a theoretical basis for understanding the molecular mechanisms underpinning radiotherapy-induced brain edema. The results of the present study may also offer potential targets for future drug development.

## Materials and methods

### Primary BMEC culture

Sprague Dawley rats (2–3 weeks old, 40–60 g body weight, male and female) were purchased from the Animal Center of Anhui Medical University, and were housed at 23 ± 2°C in separate cages with water, and fed ad libitum in a 12-h reverse light cycle. All animal experiments were performed in the department of physiology, and procedures used in the present study were conducted in accordance with the National Institutes of Health publication No. 8523 and were approved by the Animal Experimentation Ethics Committee of Anhui Medical University (No. LLSC20150048). Rats were anesthetized in a chamber containing 2% isoflurane mixed with 0.2 l/min 100% O_2_, and killed by an overdose of carbon dioxide (CO_2_), and then placed in 75% ethanol for 2 min to sterilize the body. The whole brain was removed and placed in a Petri dish containing cold phosphate-buffered saline. The cerebral hemispheres were then slowly rolled over dry filter paper to remove the pia mater and meningeal great vessels. The white matter, residual great vessels, and pia meninges were removed with fine dissection forceps, and the cerebral cortex was preserved. Next, the tissue was placed in 1 ml of Dulbecco’s modified Eagle’s medium (DMEM), and cut with ophthalmic scissors into 1-mm^3^ specimens. The tissue was then digested with 0.1% type II collagenase (containing 30 U/ml DNase I) for 1.5 h in a water bath at 37°C. After centrifugation at 1000 × ***g*** for 8 min at room temperature, the supernatant was removed. Bovine serum albumin (20%) was added, and the tissue was centrifuged again (1000 × ***g***, 20 min, 4°C) to remove the upper nervous tissue and large vessels. Collagenase/dispase (2 ml 0.1%, containing 20 U/ml DNase I) was added to the remaining precipitate, which was allowed to digestfor 1 h at 37°C in a water bath. Following centrifugation (1000 × ***g***, 8 min, room temperature), the tissue was suspended in 2 ml of DMEM and separated in a continuous gradient of 12 ml of 50% Percoll (25,000 g/l, 60 min, 4°C) with centrifugation The white and yellow layer observed near the bottom of the red blood cell layer contained the purified microvessels. After a wash, DMEM containing 20% fetal bovine serum (FBS) and 100 μg/ml heparin sodium was added to the purified microvessels, and the cells were inoculated onto a 35-mm Petri dish coated with rat tail collagen and placed in an incubator with 5% CO_2_ set at 37°C for 12–24 h. The next change of cell culture medium used fibroblast growth factor-basic (1 ng/ml) added to Medium M199.

### X-ray treatment

Rat BMECs were cultured in Medium M199 containing 10% FBS, 100 U/ml penicillin, and 100 μg/ml streptomycin in a humidified atmosphere of 5% CO_2_ at 37°C. When cell confluence reached 60%, the BMECs were irradiated with X-rays at room temperature for 8 min (2.5 Gy/min; total 20 Gy). After being cultured for an additional 24 h, the cells were used in following experiments [12,13].

### RNA extraction

The collected samples were incubated with lysis reagent for 5 min at room temperature. For every milliliter of lysis reagent, 0.2 ml of chloroform was added, and the samples were mixed using an up and down motion for 15 s and then incubated for 2–3 min at room temperature. The samples were centrifuged at 12,000 × ***g*** for 10 min at 4°C. The separated aqueous phase was transferred to a fresh tube, and 0.5 ml of isopropanol was added for a 10-min incubation at room temperature. The sample was then centrifuged at 12,000 × ***g*** for 10 min at 4°C. After being washed with 75% ethanol and centrifuged at 7500 × ***g*** for 5 min at 4°C, the supernatant was removed and air-dried at room temperature. The remaining precipitate was resuspended in 15–30 μl of diethylpyrocarbonate (DEPC)-treated water. The RNA suspension was mixed with a 1–10 part by volume of 3 M sodium acetate (pH 5.5) and 1 part by volume of isopropanol. After being incubated for 20 min at room temperature and centrifuged at 12,500 × ***g*** for 10 min at 4°C, the pellet was washed with ice-cold 70% ethanol in DEPC-treated water and centrifuged again at 10,000 × ***g*** for 5 min at 4°C. The supernatant was removed and dried at room temperature. Finally, the RNA was resuspended in DEPC-treated water. The concentration (ng/μl) and purity (absorption ratio of 260 to 230 nm) of the extracted RNA were determined using a spectrophotometer to ensure a 260/230 absorption ratio of 2.0. The RNA samples were then sequenced.

### Next-generation sequencing and data analysis

The RNA samples were sequenced using the high-throughput Illumina HiSeq 2500 sequencing platform. The sequencing length was 150 base pairs. The RNA-Seq FastQ raw data were pruned using Trimmomatic to remove adaptors and lower mass readings [14]. The quality of clean data was assessed using FastQC software [15]. The quality-approved data were mapped to the reference genome of the rat (National Center for Biotechnology Information [NCBI] genome assembly version Rnor_6.0) using HISAT2 (v2.0.13) and annotated with the annotation file (.gtf) NCBI Rnor_6.0 [16,17]. The transcript expression level was then calculated using the most common method, that is, Fragments Per Kilobase of transcript per Million fragments mapped (FPKM) [18]. The results were used to compare the differences in transcript expression among the samples. The RNA-Seq quantification software Kallisto was used to obtain a count of known mRNAs [19,20]. The differences in the number of reads were analyzed using edgR package software (http://bioconductor.org/packages/2.4/bioc/html/edgeR.html) [21]. The gene significance levels between the two groups were calculated using a negative binomial model. Gene ontology (GO) and Kyoto Encyclopedia of Genes and Genomes (KEGG) analyses were performed on differentially expressed transcriptomes, with a 2-fold change cutoff [22].

### GO and KEGG pathway enrichment analyses

The functional pathway enrichment of the proteins encoded by candidate genes were analyzed, and these genes were annotated using the Metascape database [9]. GO annotations were performed using the Metascape online tool on the screened differentially expressed transcripts. The KEGG pathway analysis of differentially expressed genes (DEGs) was also performed using the Metascape online analysis database. The DEGs that were significantly up- or down-regulated as determined using RNA-Seq data were further analyzed, and a two-sided *P* value < 0.05 was considered statistically significant.

### Protein–protein interaction (PPI) network integration

PPI analysis can provide new insights into protein function. It may also help to reveal the general organization principles of functional cellular networks. The online search tool STRING (http://string.embl.de/) was used to find interacting genes/proteins and to reveal functional relationships between proteins [23]. We constructed PPI networks of high-expression genes and of low-expression genes to predict interactions between the selected genes. Cytoscape (http://www.cytoscape.org/) is widely used to make biomolecular interaction networks into models to construct PPI networks of differentially expressed mRNAs [24]. Most previously acquired biological networks have been found to be subject to scale-free attribution [25]. Therefore, we used the molecular complex detection (MCODE) in Cytoscape software (version 3.6.0) to screen modules with MCODE scores ≥ 3 and nodes ≥ 3 in the PPI network [26]. We used topology analysis to analyze the connectivity of nodes in the PPI network so as to obtain a higher degree of important nodes (central proteins) [27]. The top 10 hub genes were selected for association. The functional enrichment analysis of individual modules was performed using Metascape, with a threshold considered statistically significant of *P*<0.05.

### Quantitative real-time polymerase chain reaction (qPCR)

We used qPCR experiment to test mRNA expression change. Briefly, cDNA was synthesized by adding total RNA into a kit of HiScript III RT SuperMix for qPCR (Vazyme, China). We mixed total RNA (4 μl), 4 × gDNA wiper Mix (4 μl) and RNase-free ddH_2_O (8 μl) together. The mixture was heated to 42°C for 2 min in a water bath. Then we added 4 μl HiScript III qRT SuperMix containing buffer, dNTP, HiScript III reverse transcriptase, RNase inhibitor and Random primers/Oligo (dT) 20VN primer mix into the heated mixture. Reaction was heated to 50°C for 15 min and then 85°C for 5 s. The Roche LightCycler 480 II PCR equipment (Roche, Switzerland) was used for qPCR reaction. Thermocycler steps were 5 min at 95°C, followed by 40 cycles of 15 s at 95°C, 30 s at 60°C. The primer sequences are listed in Table 1. The 2^−ΔΔCT^ method was used to analyze relative gene expression. *Gapdh* was used as a control.

**Table 1:** The primer sequences used in qPCR

Gene	Forward primer	Reverse primer
*Agtpbp1*	5′-TTTTGGATGAAGATGAACCTCG-3′	5′-AATCGCCTGTATTCTCCGCTA-3′
*Anln*	5′-CTCCTGGGAAGATGATGTAAGC-3′	5′-GGATTTGGATAAACAAGCGGTA-3′
*Ap3b1*	5′-CTTGGCACCTTATCTCATACTCT-3′	5′-TACTTCTACATTGCGAACCGA-3′
*Ass1*	5′-CCCAACACCCCAGATGTCCTT-3′	5′-GCGGTTCTCCACGATGTCAAT-3′
*Cttn*	5′-ACCCTGATTTTGTGAACGATG-3′	5′-GCTCCTTCTCCTTGAGTGTCTG-3′
*Dnajc27*	5′-GTATGTGCCAACAAGATTGACTG-3′	5′-ACTGCTATTTGCGGTAGGACG-3′
*Gapdh*	5′-TCTCTGCTCCTCCCTGTTC-3′	5′-ACACCGACCTTCACCATCT-3′
*Ltbp2*	5′-ACTGGGTGAACGAAGATGGC-3′	5′-GGACAGAGGCACTGGTAGGAA-3′
*Ppp1r18*	5′-AGGCTGAGAAGGAGGAGGCG-3′	5′-GGAGACAGAGGGGCTGGTGC-3′
*Prkar2b*	5′-AAGGTGGTAGATGTGATTGGC-3′	5′-CTGCTCTTGGCTTGTTAGTGA-3′
*Zfp106*	5′-AAACTCTGATGATAGGCAACCC-3′	5′-CCTCAAAGAATGTGGAAAACTG-3′

### Statistics

All data were expressed as mean ± SEM. Two-tailed Mann–Whitney *U-*test was used to determine statistical significance. Differences were considered significant with a value of *P*<0.05.

## Results

### Quality of RNA sequencing

**Figure 1:**
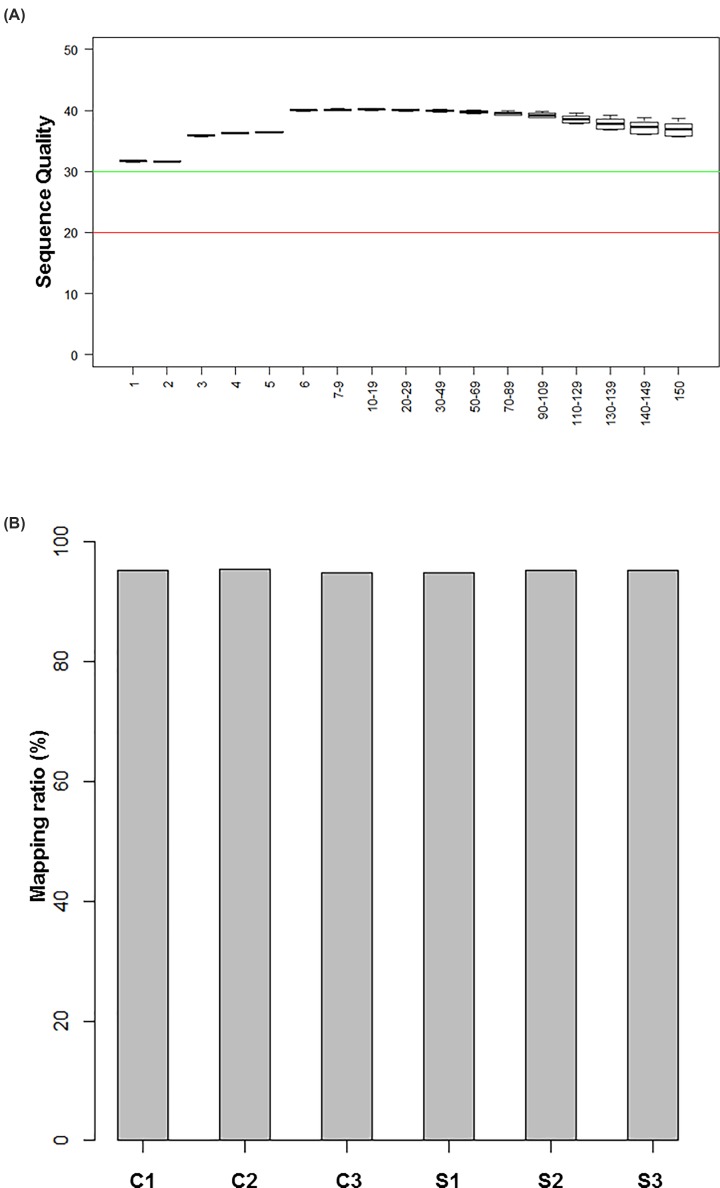
Sequencing quality (**A**) Box plot showing the sequencing quality of the six sample reads. The *X*-axis is the base position for each reading (bp indicates base pair). The *Y*-axis is the sequence quality score. A median score of less than 20 in the sequencing quality score means poor sequencing quality, and above 30 means good sequencing quality. (**B**) Mapping ratio of each sample in control (C1-C3) and X-ray–treated (S1-S3) groups. Gray bars represent samples that were sequenced. The abscissa represents the sample number and the ordinate represents the percentage of the alignment.

We sequenced and obtained raw data from six primary cultured rat BMEC samples through the Illumina platform. After trimming and filtering, the quality of data was good. The median score of the reads in all samples was above 30, and all samples passed the quality check (Figure 1A).

**Figure 2:**
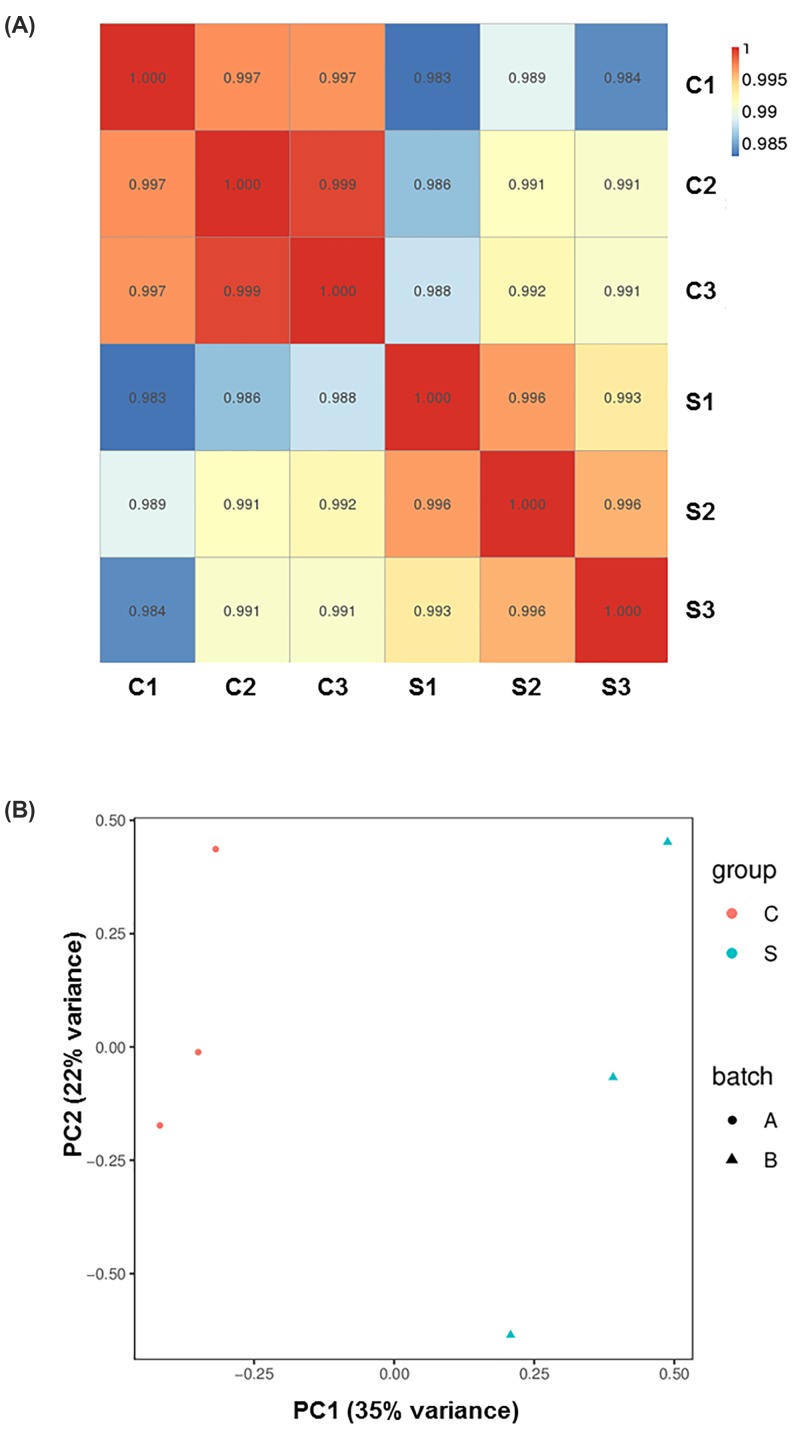
Transcript expression correlation and principal component analysis (**A**) Transcript-based sample correlation analysis showing the transcript expression level correlation between control (C1-C3) and X-ray–treated (S1-S3) groups. The abscissa indicates the sample name, the ordinate indicates the corresponding sample name, and the color indicates the correlation coefficient size. (**B**) Principal component analysis based on log2 (FPKM+1) in control (C) and X-ray–treated (S) groups. Each point in the coordinate system represents a sample, with red dots representing control (C1-C3) groups and blue triangles representing X-ray–treated (S1-S3) groups. PC1 is the main component 1, and PC2 is the main component 2. The percentage refers to the contribution of this principal component to the overall variance. The distance between the points reflects the difference between the samples they correspond to.

The trimmed high-quality reads were used for further data analysis. Mapping and quantification were conducted using the standard pipelines of HISAT2 and StringTie, which are based on the rat reference genome annotation file of Ensembl and its release 92 of Rnor_6.0 [17]. On average, the mapping ratios for all six samples were high (>90%), indicating that the reference genome was appropriate and non-contaminated (Figure 1B) [28]. We examined the rationality and experimental reliability of the sample selection by analyzing the correlation of mRNA expression levels using correlation coefficient maps among the sequenced samples (Figure 2A) and using principal component analysis (Figure 2B). The results showed that the correlations among the groups were relatively high and that the distribution of the samples was consistent with the treated groups. There was a significant difference between the treated group and the control group. The results indicated that the experimental data were of both high quality and high reliability.

### Identification of DEGs

**Figure 3:**
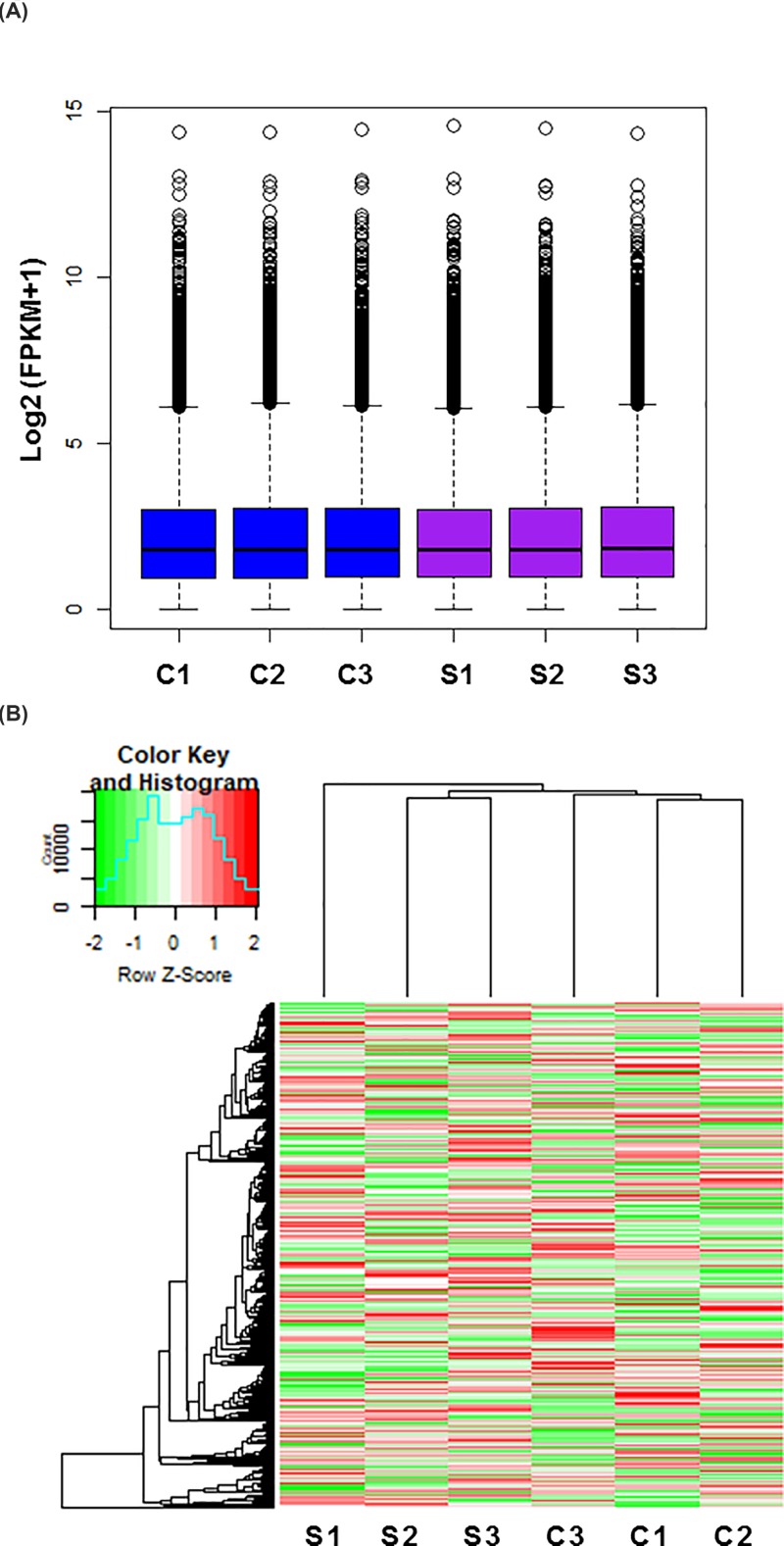
Overview of expression profiles of transcripts (**A**) Box plot showing an overview of the expression profiles of transcripts in control (C1-C3) and X-ray–treated (S1-S3) groups. *X*-axis shows the sample name; *Y*-axis is the log2 (FPKM+1) value. (**B**) Transcript-based hierarchical clustering analysis combining tissues with similar natures. Scaled to log2 (FPKM+1) expression values by different color intensities, the *Z* scores of each transcript are normalized, and green and red represent low and high expression levels, respectively.

**Figure 4:**
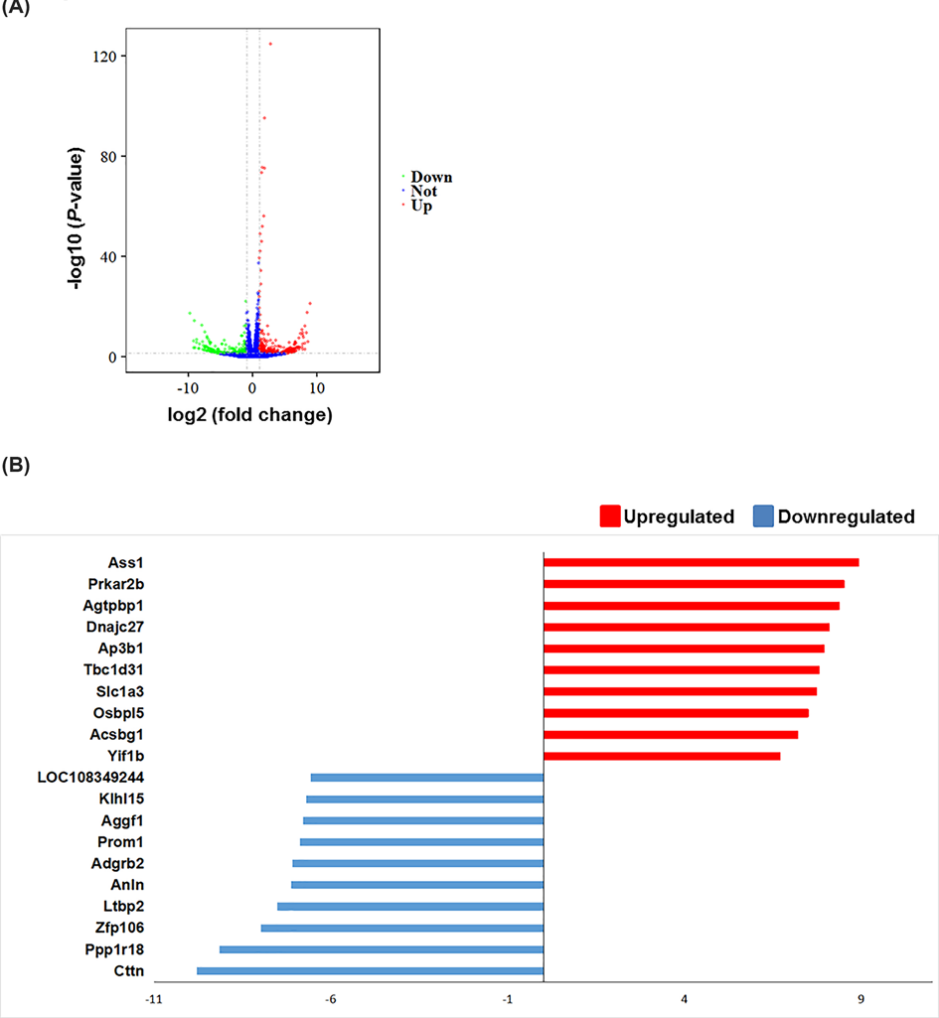
Differential expression of data between two sets of samples (**A**) Volcano map. Red points represent upregulated transcripts screened on the basis of absolute fold change ≥ 2.0 and a corrected *P* value of < 0.05. Green points represent the expression of transcripts that were down-regulated, screened on the basis of absolute fold change ≥ 2.0 and a corrected *P* value of < 0.05. The black points represent transcripts with no significant change. (**B**) The most substantially (*P*<0.05) up-regulated and down-regulated transcripts based on fold changes following irradiation of endothelial cells (*n*=3).

We investigated the overall transcript expression levels of BMECs in the control and X-ray–treated groups by using log10 (FPKM+1) values and found that the overall changes were not significantly different from the control groups (Figure 3A). Cluster analysis of the differentially expressed mRNAs showed significant differences among the groups (Figure 3B). A total of 383 genes were significantly differentially expressed, of which 183 were up- and 200 were down-regulated. The results of the differentially expressed mRNAs are displayed as a volcano map in Figure 4A. In primary cultured rat BMECs irradiated with X-rays, the top 10 up-regulated DEGs were *Ass1, Prkar2b, Agtpbp1, Dnajc27, Ap3b1, Tbc1d31, Slc1a3, Osbpl5, Acsbg1* and *Yif1b*. The top 10 down-regulated DEGs were *Cttn, Ppp1r18, Znf106, Ltbp2, Anln, Adgrb2, Prom1, Aggf1, Klhl15* and *LOC108349244* (Figure 4B).

### DEGs reflect changed biological functions

**Figure 5:**
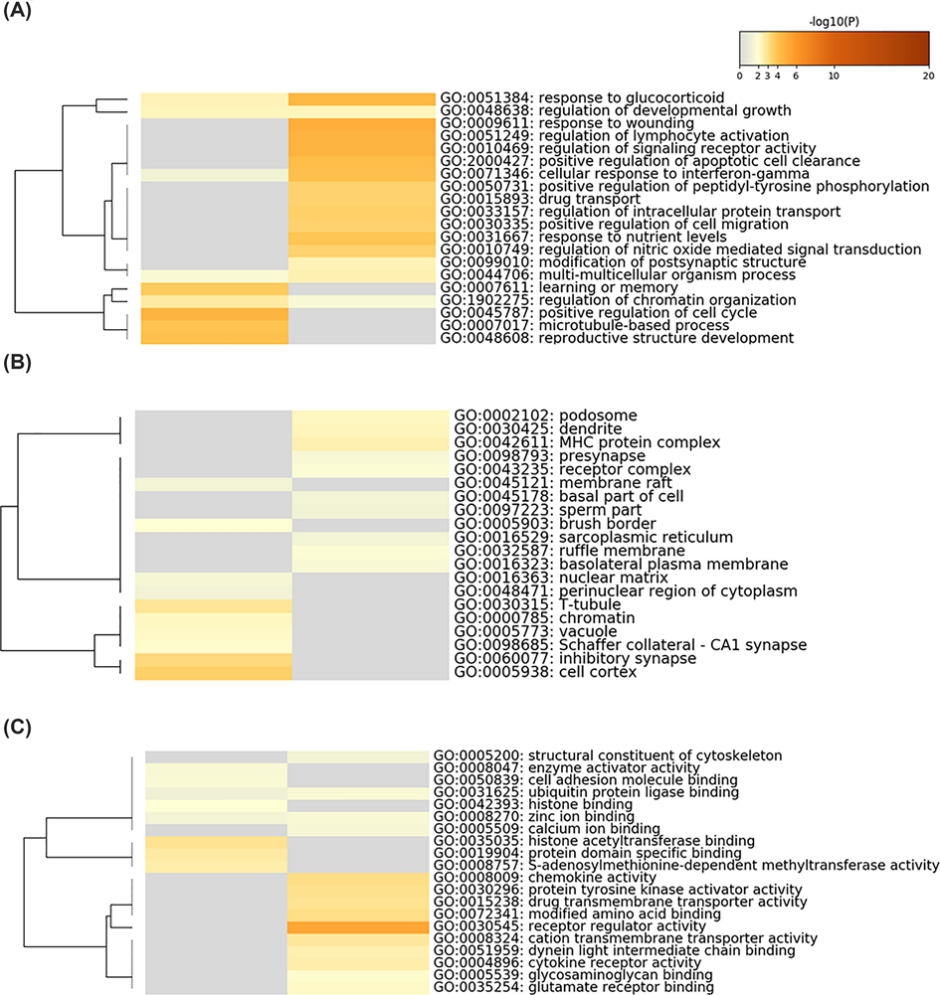
GO functional category enrichment following irradiation of endothelial cells Heat maps of the top 20 enriched (**A**) biological processes, (**B**) cell composition, and (**C**) molecular functions following irradiation of endothelial cells. The result on the left is from the down-regulated gene list, and the right is from the up-regulated gene list. The ordinate represents the corresponding Go entry. Colors represent *P* values (-log10 (*P*)).

The analysis of DEGs by GO functional annotation for the category biological process revealed that the up-regulated transcripts were mainly involved in regulation of lymphocyte activation, positive regulation of apoptotic cell clearance, positive regulation of cell migration, and response to nutrient levels. The down-regulated transcripts were primarily involved in positive regulation of cell cycle, reproductive structure development, microtubule-based process and learning or memory. Both of the up- and down-regulated transcripts were involved in response to glucocotticoid, regulation of developmental growth and cellular response to interferon-gamma (Figure 5A).

For the cellular component category, the up-regulated transcripts were mainly involved in the composition of cells in some components of the biofilm and plasma membrane protein complexes. The downregulated transcripts were mainly involved in some cell cycle–related components, such as chromosomes, telomeres, and central granules (Figure 5B).

For the category of molecular function, the up-regulated transcripts were mainly involved in receptor modulator activity, chemokine activity, drug transmembrane transporter activity, protein tyrosine kinase activator activity, cationic transmembrane transporter activity, and cytokine receptor activity. The down-regulated transcripts were primarily involved in cell adhesion molecule binding, histone acetyltransferase binding and protein domain-specific junctions, catalytic activity acting on DNA, nucleotide transferase activity, nuclear hormone receptor binding, chromatin binding, and unfolded protein binding (Figure 5C).

**Figure 6:**
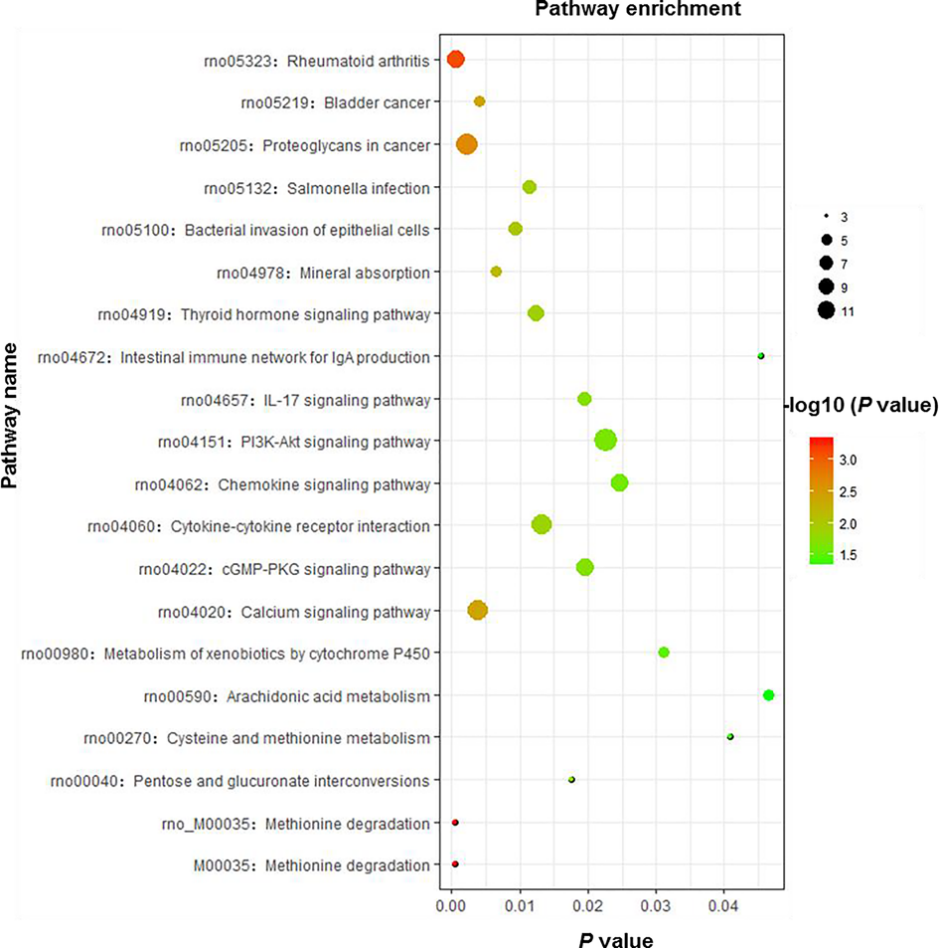
KEGG enrichment results Scatterplot of the top 20 KEGG enrichment results of differentially expressed transcripts in each pairwise comparison that were annotated in the particular pathway term. The *X*-axis indicates the rich factor, and the *Y*-axis indicates pathway. RichFactor is the ratio of differentially expressed gene numbers to all gene numbers annoted in the pathway term. A high RichFactor represents greater intensiveness. A lower *P* value represents greater intensity.

**Figure 7:**
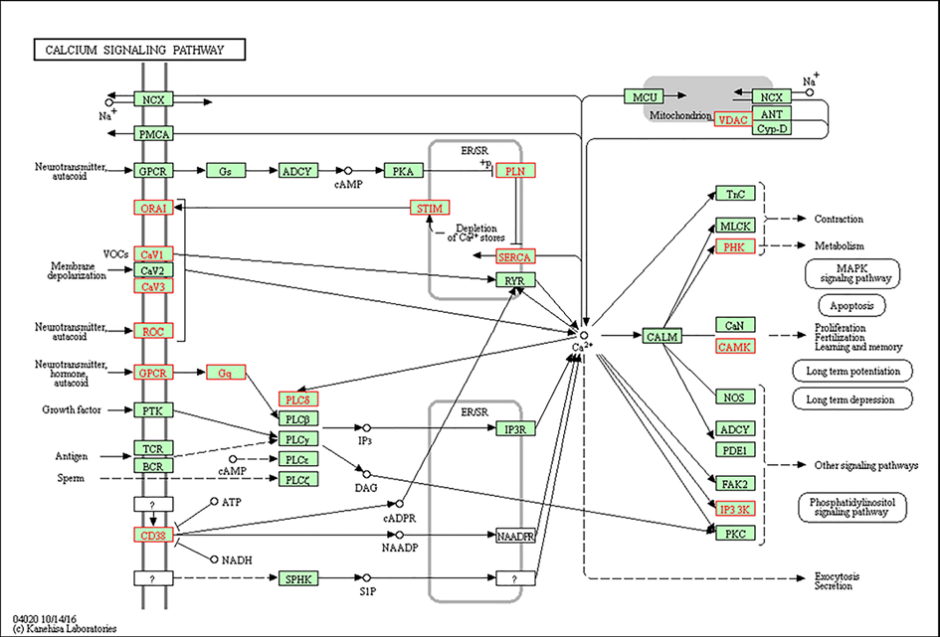
Ca^2+^ signaling pathway enrichment based on Kyoto Encyclopedia of Genes and Genomes results Red color represents a gene that is both up-regulated and involved in Ca^2+^ signaling pathway.

We found that the largest number of DEGs were related to the metabolic pathway. Interestingly, several important pathways, including the Ca^2+^ signaling pathway, phosphoinositide 3-kinase (PI3K)–Akt signaling pathway, and methionine degradation, were identified to be in the top 20 enriched pathway terms (Figure 6). Because Ca^2+^ plays an important role in endothelial cell function and vascular diseases, we examined the up-regulated transcripts that were enriched in the Ca^2+^ signaling pathway, and the results are shown in Figure 7.

### PPI network integration

**Figure 8:**
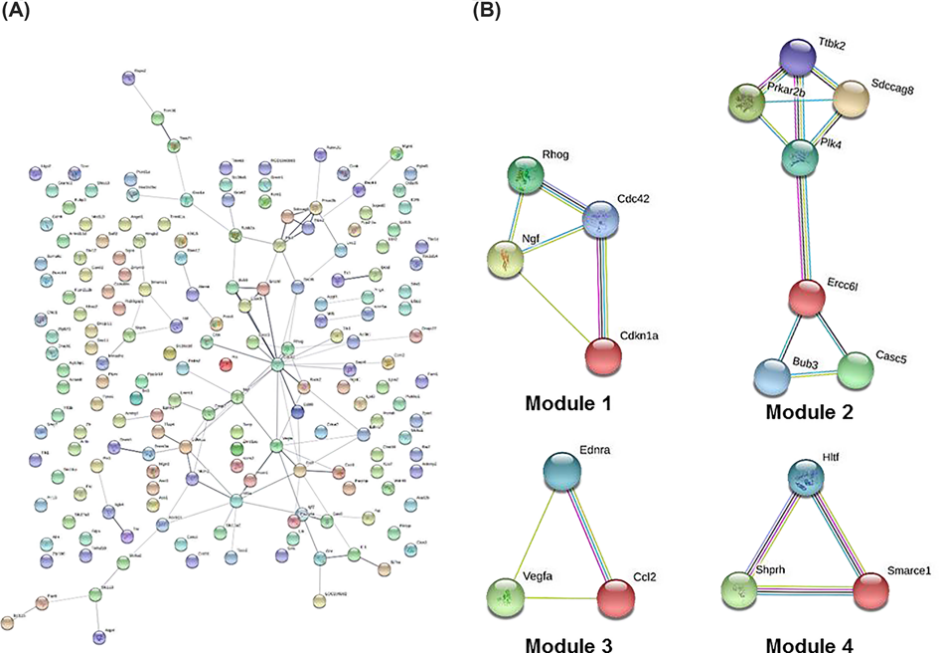
Protein–protein interaction (**A**) Interaction network and (**B**) modules. Circles represent genes, lines represent protein interactions between genes, and line colors represent evidence of interactions between proteins.

**Figure 9:**
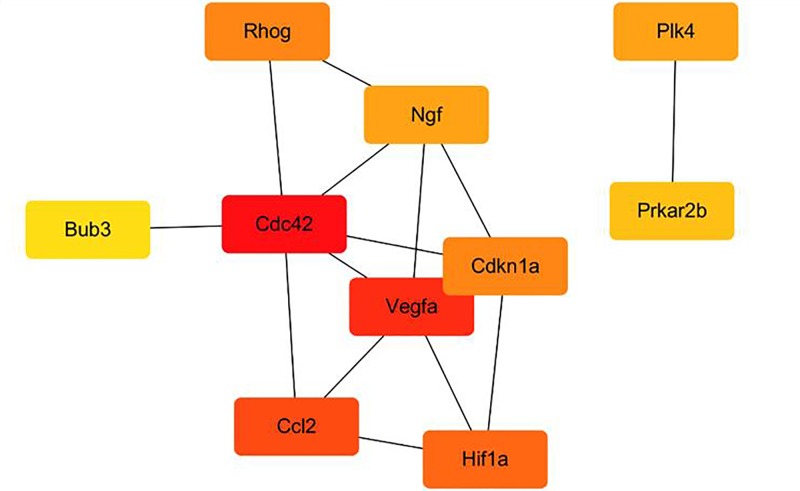
Top 10 hub genes Edges represent the protein–protein associations. Red color indicates genes with high scores. Yellow and orange color indicate genes with middle scores, increasing respectively.

We constructed a PPI network encoded by the DEGs (Figure 8A). After we analyzed the PPI network of significant differentially expressed transcripts, four modules were obtained. These genes participate in the main pathways of positively regulated cell cycle processes: DNA damage response, signal transduction by p53 class mediator resulting in cell cycle arrest, positive regulation of cell adhesion, mitotic prometaphase, positive regulation of leukocyte chemotaxis, and chromatin organization (Figure 8B) [29]. Furthermore, as shown in Figure 9, the 10 most notable genes showing significant interactions were *Cdkn1a, Ccl2, Ngf, Prkar2b, Cdc42, Hif1a, Rhog, Bub3, Vegfa*, and *Plk4*.

### Examination of top gene expression by qPCR

**Figure 10:**
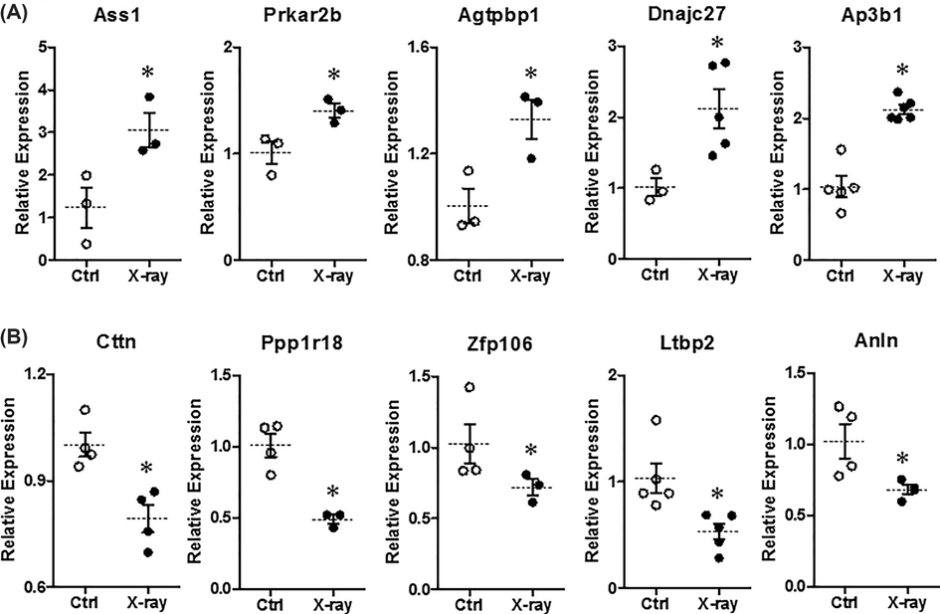
Expression changes of top five differential expression genes resolved by RNA sequence The mRNA relative expression levels of top five up-regulated (**A**) and down-regulated (**B**) genes in control (Ctrl) and X-ray–treated (X-ray, 20 Gy) brain microvascular endothelial cell of rats. Values are shown as the mean ± SEM (*n*=3–6). *P<0.05 for X-ray vs*.* Ctrl group.

To confirm the change of mRNA expression analyzed by RNA-Seq, we used qPCR to examine the mRNA expression levels of top five genes, including top five up-regulated genes (*Ass1, Prkar2b, Agtpbp1, Dnajc27, Ap3b1*) and top five down-regulated genes (*Cttn, Ppp1r18, Znf106, Ltbp2, Anln*). Our data showed that the expression levels of top five up-regulated genes (*Ass1, Prkar2b, Agtpbp1, Dnajc27, Ap3b1*) were significantly increased (Figure 10A), but top five down-regulated genes (*Cttn, Ppp1r18, Znf106, Ltbp2, Anln*) were significantly decreased (Figure 10B) in X-rays treatment group compared with control group. Our result of qPCR suggested that the findings from RNA-Seq were trustable.

## Discussion

The cause of radiation-induced brain edema varies and the related pathogenesis is complex, which makes it difficult to treat in the clinic [8]. Two main cell types are sensitive to radiation damage: oligodendrocytes and vascular endothelial cells [7]. Injury to these cell types will directly lead to the demyelination of nerve fibers and the enhancement of capillary permeability and will eventually trigger a partial collapse of the BBB system. Therefore, it is important to understand the differential expression of the genes related to the BMEC transcriptome after brain radiotherapy and the effects of this differential expression on radiation-induced brain edema.

In the present study, we found that the DEGs were associated with endothelial cell function, and the expression changes of DEGs could be confirmed by qPCR experiments. These findings may be the first evidence showing a linkage between changes in BMEC transcriptome and function after radiation exposure. Among the top 10 down-regulated transcripts, *Aggf1* encodes an angiogenic factor that promotes angiogenesis and the proliferation of endothelial cells [28,30]. The *Cttn*-encoded protein is located in the cytoplasm where the cell matrix is in contact. This protein regulates the interaction between components of the adhesive knot and is associated with cytoskeleton and cell adhesion structures essential for sphingosine-1-phosphate–mediated endothelial cell barrier enhancement [31,32]. *Prom1* encodes a pentaspan transmembrane glycoprotein. This protein related to membrane processes is commonly expressed on adult stem cells and may influence cell differentiation, proliferation, and apoptosis [33]. *Anln* encodes an actin-binding protein that plays a role in cell growth, migration, and cytokinesis. The protein is thought to regulate actin cytoskeletal dynamics [34]. The protein encoded by *Aggf1* has an effect of promoting endothelial cell proliferation, and may decrease the proliferation of BMECs when it is down-regulated after radiation irradiation [30]. Because they are involved in cytoskeletal composition and cell adhesion, the proteins encoded by *Ltbp2, Cttn, Klhl15, Prom1*, and *Anln* may change cell morphology after irradiation, resulting in decreased connectivity between cells [10,32,35–37]. Few studies have previously indicated that these genes are involved in the development and progression of radiation-induced brain edema. We believed that these genes may potentially play an important role in this process.

The results of our GO analysis suggested that radiation exposure increased apoptosis and vascular permeability, affected cell morphology, impeded the normal cell cycle, weakened cell proliferation, and impaired cell connections. All of these may affect vascular endothelial cell function and be involve in radiation-induced endothelial cell dysfunction.

The enriched KEGG pathways of the DEGs included the Ca^2+^ signaling pathway, PI3K–Akt signaling pathway, and methionine degradation. Under various pathological conditions, dysregulation of intracellular Ca^2+^ may induce sharp rises in the intracellular free Ca^2+^ ion concentration, leading to Ca^2+^ overload. Studies have shown that radiation (X-rays, gamma rays, etc.) can induce a large Ca^2+^ influx, intracellular Ca^2+^ overload, and cell dysfunction [38]. We found that irradiated BMECs showed changes in the Ca^2+^ signaling pathway, suggesting that radiation-induced intracellular Ca^2+^ dysregulation may disrupt the BBB integrity to cause or exacerbate brain edema.

We constructed and analyzed a PPI network encoded by differentially expressed transcripts. The following 10 closely related genes were identified: *Cdkn1a, Ccl2, Ngf, Prkar2b, Cdc42, Hif1a, Rhog, Bub3, Vegfa*, and *Plk4*. The proteins encoded by these genes were key nodes in the PPI network and may be the main proteins associated with brain edema. Our PPI network analysis also showed that *Prkar2b, Cdkn1a, Vegfa, Ccl2, Bub3*, and *Ngf* were up-regulated, while *Hif1a, Cdc42, Plk4*, and *Rhog* were down-regulated by radiation treatment.

There are very few reports showed the directly evidence that the *Prkar2b, Plk4*, and *Bub3* involved in BMECs damage. However, *Cdkn1a*-encoded protein plays a key role in proliferation, migration and tube formation in response to hypoxia in endothelial cells [39]. In addition, others demonstrated that CDKN1A protein can inhibit cultured BMECs apoptosis [35]. Our study showed that *Cdkn1a* mRNA was significantly increased in the radiation treatment group, which suggests that increased *Cdkn1a* expression may be protective against X-ray-induced cell injury. The *Vegfa* gene is a member of the PDGF/VEGF growth factor family [40]. The *Vegfa*-encoded protein induces proliferation and migration of vascular endothelial cells and is essential for both physiological and pathological angiogenesis. It also has anti-angiogenic and vascular permeability-inducing functions [41,42]. We believe that up-regulated VEGFA protein may lead to increased microvascular permeability in the brain. During neuroinflammation, VEGF is elevated in the central nervous system, resulting in a change in the junction complex of the BBB to cause destruction of the BBB [43]. In addition, VEGF can down-regulate the expression of claudin-5 and induce the reorganization of F-actin microfilaments, which increases the centripetal tension within the cells, leading to cell retraction [44]. *Ccl2* gene is also up-regulated by radiation treatment in BMECs. Block of CCL2 protein decreased the permeability of BBB in cultured endothelial cells during ischemia/reperfusion [36], while another study reported that CCL2 treatment alone did not disrupt the barrier sufficiently to change BBB permeability in BMECs [37]. Our data displayed the increased mRNA expression of CCL2 in the X-rays treatment group. But further study is needed to clarify the effect of CCL2 on X-ray-induced BMECs injury. In human dermal microvascular endothelial cells, one report showed that *Ngf*-encoded protein treatment enhanced cell proliferation [40], and other study showed that in hypoxic retinal endothelium, NGF attenuated cell apoptosis, and may be as a potential target for proliferative retinopathies [41]. Our data showed that *Ngf* mRNA expression was significantly up-regulated in the X-rays treatment group. Therefore, based on these evidences, we hypothesize that increased expression of NGF in the radiation treatment maybe has a protective effect on the BMECs. Moreover, one study reported that inhibition of *Hif1a*-encoded protein attenuated BBB damage in acute cerebral ischemia, and we also found that *Hif1a* mRNA was down-regulated by radiation treatment in the present study [42], while deficiency of *Cdc42*-encoded protein also displayed increased vascular permeability *in vivo* and activation of CDC42 might protect endothelial barrier [43,45]. Moreover, it is interesting that one study showed that *Rhog*-encoded protein played an essential role of angiogenesis in vascular endothelial cells via mediating the CDC42 [46]. Whereas we found that both *Cdc42* and *Rhog* genes were down-regulated by the radiation treatment in the present study. Thus, the reduction of *Cdc42* and *Rhog* genes expression may be one reason for X-ray-induced endothelial cell injury.

## Conclusion

In summary, we used next-generation sequencing technology to determine the expression profile of the transcriptome in rat BMECs after radiation exposure to explore the mechanism underlying radiotherapy-induced BMEC damage. Our bioinformatics analysis revealed DEGs and changed signaling pathways in BMECs after radiation exposure and their significance in radiation-induced brain edema. These results point to several specific genes as being potential candidates of the molecular mechanisms underpinning radiation-induced brain edema and provide new potential targets for clinical treatment and prevention of radiation-induced cerebral edema.

## Abbreviations

BBB: blood–brain barrier
BMEC: brain microvascular endothelial cell
DEGs: differentially expressed genes
DEPC: diethylpyrocarbonate
MCODE: molecular complex detection
PI3K: phosphoinositide 3-kinase
PPI: protein–protein interaction
